# Association between multiple comorbidities and self-rated health status in middle-aged and elderly Chinese: the China Kadoorie Biobank study

**DOI:** 10.1186/s12889-018-5632-1

**Published:** 2018-06-15

**Authors:** Xingyue Song, Jing Wu, Canqing Yu, Wenhong Dong, Jun Lv, Yu Guo, Zheng Bian, Ling Yang, Yiping Chen, Zhengming Chen, An Pan, Liming Li, on behalf of the China Kadoorie Biobank Collaborative Group, Junshi Chen, Zhengming Chen, Rory Collins, Liming Li, Richard Peto, Daniel Avery, Ruth Boxall, Derrick Bennett, Yumei Chang, Yiping Chen, Zhengming Chen, Robert Clarke, Huaidong Du, Simon Gilbert, Alex Hacker, Mike Hill, Michael Holmes, Andri Iona, Christiana Kartsonaki, Rene Kerosi, Ling Kong, Om Kurmi, Garry Lancaster, Sarah Lewington, Kuang Lin, John McDonnell, Iona Millwood, Qunhua Nie, Jayakrishnan Radhakrishnan, Sajjad Rafiq, Paul Ryder, Sam Sansome, Dan Schmidt, Paul Sherliker, Rajani Sohoni, Becky Stevens, Iain Turnbull, Robin Walters, Jenny Wang, Lin Wang, Neil Wright, Ling Yang, Xiaoming Yang, Zheng Bian, Yu Guo, Xiao Han, Can Hou, Jun Lv, Pei Pei, Yunlong Tan, Canqing Yu, Zengchang Pang, Ruqin Gao, Shanpeng Li, Shaojie Wang, Yongmei Liu, Ranran Du, Yajing Zang, Liang Cheng, Xiaocao Tian, Hua Zhang, Yaoming Zhai, Feng Ning, Xiaohui Sun, Feifei Li, Silu Lv, Junzheng Wang, Wei Hou, Mingyuan Zeng, Ge Jiang, Xue Zhou, Liqiu Yang, Hui He, Bo Yu, Yanjie Li, Qinai Xu, Quan Kang, Ziyan Guo, Dan Wang, Ximin Hu, Hongmei Wang, Jinyan Chen, Yan Fu, Zhenwang Fu, Xiaohuan Wang, Min Weng, Zhendong Guo, Shukuan Wu, Yilei Li, Huimei Li, Zhifang Fu, Ming Wu, Yonglin Zhou, Jinyi Zhou, Ran Tao, Jie Yang, Jian Su, Fang Liu, Jun Zhang, Yihe Hu, Yan Lu, Liangcai Ma, Aiyu Tang, Shuo Zhang, Jianrong Jin, Jingchao Liu, Zhenzhu Tang, Naying Chen, Ying Huang, Mingqiang Li, Jinhuai Meng, Rong Pan, Qilian Jiang, Jian Lan, Yun Liu, Liuping Wei, Liyuan Zhou, Ningyu Chen, Ping Wang, Fanwen Meng, Yulu Qin, Sisi Wang, Xianping Wu, Ningmei Zhang, Xiaofang Chen, Weiwei Zhou, Guojin Luo, Jianguo Li, Xiaofang Chen, Xunfu Zhong, Jiaqiu Liu, Qiang Sun, Pengfei Ge, Xiaolan Ren, Caixia Dong, Hui Zhang, Enke Mao, Xiaoping Wang, Tao Wang, Xi Zhang, Ding Zhang, Gang Zhou, Shixian Feng, Liang Chang, Lei Fan, Yulian Gao, Tianyou He, Huarong Sun, Pan He, Chen Hu, Xukui Zhang, Huifang Wu, Pan He, Min Yu, Ruying Hu, Hao Wang, Yijian Qian, Chunmei Wang, Kaixu Xie, Lingli Chen, Yidan Zhang, Dongxia Pan, Qijun Gu, Yuelong Huang, Biyun Chen, Li Yin, Huilin Liu, Zhongxi Fu, Qiaohua Xu, Xin Xu, Hao Zhang, Huajun Long, Xianzhi Li, Libo Zhang, Zhe Qiu

**Affiliations:** 1 Department of Epidemiology and Biostatistics, and Ministry of Education Key Laboratory of Environment and Health, and State Key Laboratory of Environmental Health (Incubating), School of Public Health, Tongji Medical College, Huazhong University of Science and Technology, 13 Hangkong Rd, Wuhan, 430030 China; 2 Department of Epidemiology and Biostatistics, School of Public Health, Peking University Health Science Center, 38 Xueyuan Rd, Beijing, 100191 China; 3 Chinese Academy of Medical Sciences, Dong Cheng District, Beijing, China; 4 Clinical Trial Service Unit and Epidemiological Studies Unit (CTSU), Nuffield Department of Population Health, University of Oxford, Oxford, UK

**Keywords:** Comorbidity, Self-rated health status, Cross-sectional study, Chinese population

## Abstract

**Background:**

Understanding the correlates of self-rated health (SRH) can help public health professionals prioritize health-promotion and disease-prevention interventions. This study aimed to investigate the association between multiple comorbidities and global SRH and age-comparative SRH.

**Methods:**

A total of 512,891 participants aged 30–79 years old were recruited into the China Kadoorie Biobank study from ten regions between 2004 and 2008. Multivariate logistic regression models were used to estimate the odds ratios (ORs) for the associations between comorbidities (including diabetes, hypertension, coronary heart disease, rheumatic heart disease, stroke, tuberculosis, emphysema/bronchitis, asthma, cirrhosis/chronic hepatitis, peptic ulcer, gallbladder disease, kidney disease, fracture, rheumatic arthritis, psychiatric disorders, depressive symptoms, neurasthenia, head injury and cancer) and SRH. Population attributable risks (PARs) were used to estimate the contribution of multiple comorbidities to poor global SRH and worse age-comparative SRH.

**Results:**

After adjusting for covariates, suffering from various diseases increased the chance of reporting a poor global SRH [OR (95% CI) ranged from 1.10 (1.07, 1.13) for fracture to 3.21 (2.68, 3.83) for rheumatic heart disease] and a worse age-comparative SRH [OR (95% CI) ranged from 1.18 (1.13, 1.23) for fracture to 7.56 (6.93, 8.25) for stroke]. From the population perspective, 20.23% of poor global SRH and 45.12% of worse age-comparative SRH could attributed to the cardiometabolic diseases, with hypertension (7.84% for poor global SRH and 13.79% for worse age-comparative SRH), diabetes (4.35% for poor global SRH and 10.71% for worse age-comparative SRH), coronary heart disease (4.44% for poor global SRH and 9.51% for worse age-comparative SRH) and stroke (3.20% for poor global SRH and 10.19% for worse age-comparative SRH) making the largest contribution.

**Conclusions:**

Various diseases were major determinants of global and age-comparative SRH, and cardiometabolic diseases had the strongest impact on both global SRH and age-comparative SRH at the population level. Prevention measures concentrated on these conditions would greatly reduce the total burden of poor SRH and its consequences such as poor quality of life and use of health care services.

## Background

Self-rated health (SRH), a common but comprehensive measure of a person’s global (physical and mental) health status, has been widely used to describe the health status of an individual and a population in epidemiology surveys because of the easily collected data [1]. Respondents are asked to rate their own health status and there are three types of SRH that are frequently assessed in different studies. The most commonly used one is global SRH, in which respondents are requested to rate their current overall health status on a four- or five-point scale ranging from “very good (excellent)” to “very poor” [2]. In the age-comparative SRH, respondents are asked to rate their health status as better, the same, or worse compared with other people of their ages [3]. As for time-comparative SRH, respondents are asked to compare their present health to the health status a year earlier [4]. SRH is one of the indicators recommended for health monitoring by the World Health Organization [5], and has been demonstrated to be a powerful predictor of morbidity and mortality in different populations [2, 6, 7].

Understanding the determinants and correlates of SRH can help public health professionals prioritize the interventions for health promotion and disease prevention. Previous studies have found a wide range of determinants of SRH, including sociodemographic factors, lifestyle factors, physical illness and psychological factors [8–12]. It is widely acknowledged that physical and mental illnesses are major determinants of SRH; however, few studies have comprehensively examined the relations of multiple physical and mental conditions with SRH. Many studies combined various physical illnesses into one variable to explore the association between the presence and/or number of chronic diseases and SRH [9, 13–16]. A few studies examined different chronic diseases as independent variables, but the number of diseases was limited and generally less than eight [17–20]. Only one study conducted in western Sweden has examined eleven chronic diseases and their associations with SRH, but only global SRH was used and the sample size was small (*n* = 6061) [21].

Using data from the China Kadoorie Biobank (CKB) with over half million participants, we aimed to investigate the relations of multiple comorbidities with global SRH and age-comparative SRH among middle-aged and elderly population in China.

## Methods

### Study population

The CKB study is a general population-based prospective cohort study that was designed to investigate the complex interplay of lifestyle behavior, environmental and genetic factors and risks of various chronic diseases. The detailed study design, sampling strategy and features of the study participants are previously reported [22, 23]. In brief, a total of 512,891 participants aged from 30 to79 years old were recruited from ten regions covering five rural areas (Gansu, Henan, Zhejiang, Sichuan, and Hunan provinces) and five urban cities (Harbin, Qingdao, Suzhou, Liuzhou, and Haikou) in China between 2004 and 2008. The study sites were selected based on population stability, geographic area, quality of death and disease registries, regional patterns of diseases and exposure to specific risk factors, and local commitment and capacity.

In the baseline survey, trained interviewers conducted a standardized questionnaire using a laptop-based direct data-entry system for the questionnaires, with built-in functions to avoid data missing and logical errors. The questionnaires covered general demographic characteristics, socio-economic status, diet, general health, physical activity, family medical history and mental health. After the interview, a series of physical measurements including weight, height, waist and hip circumference and blood pressures were recorded for each participant by trained technicians, and a 10-mL non-fasting blood sample was taken.

We excluded 2 participants with missing values of body weight or height. A total of 512,889 participants (40.99% male and 59.01% female) remained in the current analysis.

### Exposure variables

Physician diagnosed disease status was enquired in baseline questionnaire. Respondents were asked: “Has a doctor ever told you that you had the following disease?” followed by a list of diseases including diabetes, hypertension, coronary heart disease (CHD), stroke, rheumatic heart disease, tuberculosis, emphysema/bronchitis, asthma, cirrhosis/chronic hepatitis, peptic ulcer, gallstone/gallbladder disease, kidney disease, fracture, rheumatic arthritis, psychiatric disorders, neurasthenia, head injury and cancer. If someone reported that he/she was diagnosed with cancer, the site of cancer was also asked. Prevalent diabetes was defined if the participant met one of the following criteria [24]: 1) self-reported diagnosis of diabetes mellitus; 2) use of anti-diabetic medications; 3) fasting plasma glucose ≥7.0 mmol/L; and 4) random blood glucose ≥11.1 mmol/L. Prevalent hypertension was defined if the participants met one of the following criteria [25]: 1) systolic blood pressure of ≥140 mmHg; 2) diastolic blood pressure of ≥90 mmHg; 3) self-reported diagnosis of hypertension; and 4) use of antihypertensive medication. If someone reported that he/she had been diagnosed with a disease, the age at first diagnosis and basic treatment information was also asked. Past year major depression was assessed using the Chinese version of computerized Composite International Diagnostic Inventory — short form (CIDI-SF), and the details were described elsewhere [26].

### Outcome variables

To assess SRH status of each participant, two questions were asked in baseline interview: 1) How is your current general health status: excellent, good, fair, or poor? 2) How is your current health status compared with someone of your own age: better, about the same, worse, or don’t know? We considered the first question as global SRH and the second one as age-comparative SRH. No data were missing for two SRH variables. We categorized the global SRH into two categories to be comparable to the literature on this topic: good (excellent, good) and poor (fair, poor). For the age-comparative SRH, participants answering “don’t know” (*n* = 15,691, 3.06%) were excluded from the analysis. To be consistent with global SRH, participants answering “about the same” (*n* = 321,218, 62.63%) were excluded in the main analysis and thus age-comparative SRH was categorized into two groups: better and worse. In order to ensure the accuracy and robustness of the results, we also conducted sensitivity analysis of including participants who reported “about the same” and thus age-comparative SRH was categorized into three groups: better, about the same, and worse.

### Confounding factors

Other variables included age, sex, study location (10 regions), marital status (married, widowed, separated/divorced, never married), education level (no formal education, primary, middle or high school, college/university or higher), annual household income (< 10,000, 10,000–19,999, 20,000–34,999, ≥35,000 RMB), employment status (employed, unemployed and retired), cigarette smoking (never, former, occasionally, current smoker) and alcohol drinking (never, former, occasionally, current drinker) were also inquired in the baseline questionnaires. Body mass index (BMI) was calculated as weight divided by the square of height (kg/m^2^). The physical activity level was measured by adding up metabolic equivalent tasks (METs) for daily work or leisure activities. All participants were also inquired about their family history of five diseases, including diabetes, heart attack, stroke, mental disorders and cancer.

### Statistical analysis

Baseline demographic, socioeconomic, behavioral characteristics by global SRH categories and age-comparative SRH groups were presented by unadjusted proportions for categorical variables and unadjusted means with standard deviations (SD) for continuous variables, and compared using ANOVA and Chi-square tests for continuous and categorical variables, respectively. Logistic regression models were used to estimate odds ratios (ORs) and 95% confidence intervals (CIs) for relations of multiple comorbidities with global and age-comparative SRH. We adjusted for potential confounders in sequential steps: model 1 adjusted for sociodemographic factors (age in continuous variable, sex, study location, marital status, education level, household income and occupation in categorical variable); model 2 further adjusted for lifestyle factors (BMI in continuous variable, cigarette smoking, alcohol drinking, physical activity level and family history of five diseases, in categories); model 3 additionally adjusted for all comorbidities in the same model to test the independent associations. In the sensitivity analysis of including participants answering “about the same” for the age-comparative SRH, multinomial logistic regression models were used.

Population attributable risks (PARs) were used to estimate the proportion of the total prevalence of poor global SRH (or worse age-comparative SRH) that could be attributed to multiple comorbidities, and were calculated for each disease using the following formula: PAR=(p(OR-1))/(p(OR-1)+1) [27], where p denotes the proportion of participants with the condition in the study population, and OR is the corresponding odds ratio observed in our study.

Stratified analyses were performed according to sex, administrative regions (urban and rural), age (30–59, ≥60 years old), education level (no formal education, primary school, middle school or higher) and household income (< 10,000, 10,000–34,999 and ≥ 35,000 RMB). Tests for interaction were conducted by adding interaction terms of the certain disease and the stratifying variable in the final model. We also analyzed the impact of the duration of disease diagnosis, treatment, the number of diseases and the site of cancer on global and age-comparative SRH. Furthermore, to test the influence of undiagnosed diseases on the associations, we used hypertension and diabetes as examples in a sensitivity analysis. In the main analysis, the definitions of hypertension and diabetes included both the self-reported physician-diagnosed diseases and screening-detected diseases from the physical examination (which could be considered as undiagnosed cases). In the sensitivity analysis, we only used self-reported physician-diagnosed hypertension and diabetes (yes vs. no) as the exposures and evaluated their associations with SRH. All analyses were performed using SAS 9.3 (SAS Institute Inc), and two-sided *P* values < 0.05 were considered as statistical significance.

## Results

The baseline characteristics of participants are presented in Table 1. Of the 512,889 participants, the mean age (standard deviation, SD) was 51.53 (10.68), and 59.01% (*n* = 302,631) were women. Most participants were married (90.59%) and more than half (55.90%) were recruited from rural areas; two-thirds of the participants were employed (68.74%), and about one-fourth (26.43%) were current smokers and 17.13% were current regular drinkers. The mean (SD) MET and BMI of the participants was 21.08 (13.87) and 23.66 (3.38), respectively.

**Table 1:** Characteristics of the study participants by self-rated health status^a^

Variable	Total (*n* = 512,889)	Global Self-rated Health			Age-comparative Self-rated Health			
		Good (*n* = 234,699)	Fair/Poor (*n* = 278,190)	*P* Value^b^	Better (*n* = 93,235)	Same (n = 321,218)	Worse (*n* = 82,745)	*P* Value^b^
Age, mean (SD), y	51.53 (10.68)	50.27 (10.43)	52.59 (10.77)	< 0.001	51.14 (10.78)	51.27 (10.60)	52.45 (10.67)	< 0.001
Female gender	302,631 (59.01)	131,178 (55.89)	171,453 (61.63)	< 0.001	50,321 (53.97)	188,751 (58.76)	53,568 (64.74)	< 0.001
Physical activity, mean (SD), MET h/wk	21.08 (13.87)	22.89 (14.18)	19.55 (13.42)	< 0.001	21.60 (13.73)	21.60 (13.97)	19.08 (13.58)	< 0.001
BMI, mean (SD)	23.66 (3.38)	23.76 (3.24)	23.57 (3.49)	< 0.001	24.06 (3.22)	23.59 (3.32)	23.52 (3.73)	< 0.001
Rural residence	286,703 (55.90)	129,793 (55.30)	156,910 (56.40)	< 0.001	41,771 (44.80)	193,272 (60.17)	46,787 (56.64)	< 0.001
Married currently	464,607 (90.59)	216,143 (92.09)	248,464 (89.31)	< 0.001	84,713 (90.86)	292,908 (91.19)	73,271 (88.55)	< 0.001
Educational level^d^				< 0.001				< 0.001
No formal school	95,220 (18.57)	42,999 (18.32)	52,221 (18.77)		13,235 (14.20)	59,244 (18.44)	19,440 (23.49)	
Primary School	165,216 (32.21)	70,612 (30.09)	94,604 (34.01)		24,664 (26.45)	108,479 (33.77)	27,045 (32.68)	
Middle or high School	222,439 (43.37)	105,233 (44.84)	117,206 (42.13)		46,579 (49.96)	136,627 (42.53)	32,654 (39.46)	
College or university	30,014 (5.85)	15,855 (6.76)	14,159 (5.09)		8757 (9.39)	16,868 (5.25)	3606 (4.36)	
Annual income, Chinese Yuan^d^				< 0.001				< 0.001
0–9999	144,832 (28.24)	56,688 (24.15)	88,144 (31.68)		25,112 (26.93)	83,500 (25.99)	32,365 (39.11)	
10,000–19,999	149,013 (29.05)	64,685 (27.56)	84,328 (30.31)		26,804 (28.75)	94,432 (29.40)	23,573 (28.49)	
20,000–34,999	126,719 (24.71)	62,152 (26.48)	64,567 (23.21)		22,374 (24.00)	84,697 (26.37)	16,195 (19.57)	
> 35,000	92,325 (18.00)	51,174 (21.80)	41,151 (14.79)		18,945 (20.32)	58,589 (18.24)	10,612 (12.82)	
Employment status				< 0.001				< 0.001
Retired	84,939 (16.56)	33,170 (14.13)	51,769 (18.61)		17,709 (18.99)	49,313 (15.35)	13,997 (16.92)	
Employed	352,574 (68.74)	173,032 (73.73)	179,542 (64.54)		65,553 (70.31)	226,383 (70.48)	52,381 (63.30)	
Unemployed	75,376 (14.70)	28,497 (12.14)	46,879 (16.85)		9973 (10.70)	45,522 (14.17)	16,367 (19.78)	
Current smoker	135,554 (26.43)	67,957 (28.95)	67,597 (24.30)	< 0.001	27,937 (29.96)	85,405 (26.59)	18,733 (22.64)	< 0.001
Current alcohol drinker	87,847 (17.13)	46,470 (19.80)	41,377 (14.87)	< 0.001	21,328 (22.88)	53,474 (16.65)	10,975 (13.26)	< 0.001
Family history of five diseases^c^	197,147 (38.44)	89,283 (45.29)	107,864 (54.71)	< 0.001	39,772 (42.66)	116,535 (36.28)	35,936 (43.43)	< 0.001
Cardiometabolic diseases								
Coronary heart disease	15,472 (3.02)	3181 (1.36)	12,291 (4.42)	< 0.001	1605 (1.72)	7622 (2.37)	5861 (7.08)	< 0.001
Rheumatic heart disease	938 (0.18)	152 (0.06)	786 (0.28)	< 0.001	69 (0.07)	361 (0.11)	482 (0.58)	< 0.001
Stroke	8884 (1.73)	1612 (0.69)	7272 (2.61)	< 0.001	723 (0.78)	3608 (1.12)	4312 (5.21)	< 0.001
Hypertension	174,480 (34.02)	72,583 (30.93)	101,897 (36.63)	< 0.001	28,007 (30.34)	108,330 (33.72)	32,531 (39.31)	< 0.001
Diabetes	30,300(5.91)	9159(3.90)	21,141(7.60)	< 0.001	3709(3.98)	16,453(5.12)	9018(10.90)	< 0.001
Respiratory diseases								
Tuberculosis	7660 (1.49)	2535 (1.08)	5125 (1.84)	< 0.001	1255 (1.35)	4209 (1.31)	1996 (2.41)	< 0.001
Emphysema/bronchitis	13,289 (2.59)	3242 (1.38)	10,047 (3.61)	< 0.001	1291 (1.38)	6276 (1.95)	5233 (6.32)	< 0.001
Asthma	2806 (0.55)	695 (0.30)	2111 (0.76)	< 0.001	278 (0.30)	1322 (0.41)	1127 (1.36)	< 0.001
Musculoskeletal diseases								
Fracture	35,448 (6.91)	16,416 (6.99)	19,032 (6.84)	0.0312	6711 (7.20)	21,213 (6.60)	6377 (7.71)	< 0.001
Rheumatoid arthritis	10,626 (2.07)	2928 (1.25)	7698 (2.77)	< 0.001	1312 (1.41)	5318 (1.66)	3467 (4.19)	< 0.001
Mental diseases								
Depressive symptoms	3281 (0.64)	704 (0.30)	2577 (0.93)	< 0.001	319 (0.34)	1375 (0.43)	1502 (1.82)	< 0.001
Psychiatric disorders	1906 (0.37)	512 (0.22)	1394 (0.50)	< 0.001	145 (0.16)	868 (0.27)	815 (0.98)	< 0.001
Neurasthenia	5699 (1.11)	1503 (0.64)	4196 (1.51)	< 0.001	673 (0.72)	2702 (0.84)	2208 (2.67)	< 0.001
Digestive diseases								
Cirrhosis/chronic hepatitis	6195 (1.21)	2098 (0.89)	4097 (1.47)	< 0.001	680 (0.73)	3499 (1.09)	1707 (2.06)	< 0.001
Peptic ulcer	20,017 (3.90)	6666 (2.84)	13,351 (4.80)	< 0.001	2869 (3.08)	10,818 (3.37)	5610 (6.78)	< 0.001
Gallstone/gallbladder disease	31,001 (6.04)	10,862 (4.63)	20,139 (7.24)	< 0.001	4327 (4.64)	17,064 (5.31)	8685 (10.50)	< 0.001
Cancer	2577 (0.50)	567 (0.24)	2010 (0.72)	< 0.001	217 (0.23)	1112 (0.35)	1174 (1.42)	< 0.001
Other diseases								
Kidney disease	7577 (1.48)	2401 (1.02)	5176 (1.86)	< 0.001	1133 (1.22)	3664 (1.14)	2574 (3.11)	< 0.001
Head injury	5653 (1.10)	2335 (0.99)	3318 (1.19)	< 0.001	1029 (1.10)	3182 (0.99)	1272 (1.54)	< 0.001

*Abbreviations*: *BMI* body mass index, *MET* metabolic equivalent

^a^Data are presented as frequency (percentage) unless otherwise indicated

^b^Two-sided *P* values were derived from ANOVA for continuous variables and Chi-square test for categorical variables

^c^Including family history of diabetes, heart attack, stroke, mental disorders and cancer

^d^The percentages may not add up to 100% because of rounding

The most prevalent disease was hypertension (34.02%), followed by fracture (6.91%), gallstone/gallbladder disease (6.04%), diabetes (5.91%), peptic ulcer (3.90%), CHD (3.02%), emphysema/bronchitis (2.59%), rheumatoid arthritis (2.07%), stroke (1.73%), cirrhosis/chronic hepatitis (1.21%), tuberculosis (1.49%), kidney disease (1.48%), neurasthenia (1.11%), head injury (1.10%), depressive symptoms (0.64%), asthma (0.55%), cancer (0.50%), psychiatric disorders (0.37%) and rheumatic heart disease (0.18%) (Table 1).

Table 2 shows the associations between various diseases and global SRH. In the final multivariate model, suffering from any of the diseases increased the chance of reporting a poor global SRH, the OR (95% CI) ranged from 1.10 (1.07, 1.13) for fracture to 3.21 (2.68, 3.83) for rheumatic heart disease. Similar findings were obtained in the stratified analyses, while the magnitude of the association between some diseases and global SRH was significantly stronger in one stratum than the other (Appendix Tables 5–7).

**Table 2:** Multiple comorbidities associated with global self-rated health status

	Model 1^a^		Model 2^b^		Model 3^c^	
	OR	95% CI	OR	95% CI	OR	95% CI
Cardiometabolic diseases						
Coronary heart disease	2.91	2.79, 3.03	2.87	2.75, 2.99	2.54	2.43, 2.65
Rheumatic heart disease	3.74	3.14, 4.46	3.59	3.01, 4.28	3.21	2.68, 3.83
Stroke	3.30	3.12, 3.49	3.10	2.93, 3.28	2.91	2.74, 3.07
Hypertension	1.24	1.22, 1.26	1.28	1.26, 1.29	1.25	1.23, 1.26
Diabetes	1.86	1.81, 1.91	1.85	1.80, 1.89	1.77	1.72, 1.82
Respiratory diseases						
Tuberculosis	1.58	1.50, 1.66	1.54	1.46, 1.61	1.48	1.41, 1.56
Emphysema/bronchitis	2.65	2.55, 2.77	2.57	2.46, 2.67	2.47	2.37, 2.58
Asthma	2.55	2.33, 2.78	2.48	2.27, 2.71	2.05	1.87, 2.24
Musculoskeletal diseases						
Fracture	1.11	1.08, 1.14	1.12	1.09, 1.15	1.10	1.07, 1.13
Rheumatoid arthritis	1.99	1.90, 2.08	1.99	1.90, 2.08	1.83	1.75, 1.92
Mental disease						
Depressive Symptoms	3.09	2.83, 3.36	3.01	2.76, 3.28	2.66	2.44, 2.90
Psychiatric disorders	2.52	2.27, 2.79	2.45	2.20, 2.71	2.16	1.94, 2.40
Neurasthenia	2.52	2.37, 2.67	2.47	2.33, 2.63	2.14	2.01, 2.27
Digestive diseases						
Cirrhosis/chronic hepatitis	1.80	1.71, 1.90	1.72	1.63, 1.82	1.67	1.58, 1.77
Peptic ulcer	1.78	1.72, 1.83	1.72	1.67, 1.78	1.69	1.64, 1.75
Gallstone/gallbladder disease	1.69	1.65, 1.73	1.68	1.63, 1.72	1.59	1.55, 1.64
Cancer	2.65	2.41, 2.91	2.43	2.21, 2.67	2.56	2.33, 2.82
Other diseases						
Kidney disease	1.81	1.72, 1.90	1.78	1.70, 1.88	1.63	1.55, 1.72
Head injury	1.23	1.17, 1.30	1.24	1.17, 1.31	1.19	1.12, 1.26

*Abbreviations*: *CI* confidence intervals, *OR* odds ratio

^a^Model 1: adjusted for age, sex, study location, marital status, education level, income and occupation

^b^Model 2: model 1 plus cigarette smoking, alcohol drinking, physical activity, BMI and family history of five disease (stroke, heart attack, diabetes, mental disorders and cancer)

^c^Model 3: model 2 plus all comorbidities in the same mode

Table 3 shows the associations between various diseases and age-comparative SRH. In the final multivariate model, similarly, all diseases were associated with higher odds of a worse age-comparative SRH: the OR (95% CI) ranged from 1.18 (1.13, 1.23) for fracture to 7.56 (6.93, 8.25) for stroke. Similar findings were obtained in the stratified analyses (Appendix Tables 8–10). Stroke remained to have the strongest association in certain subgroups (rural residence, male, age subgroups, all education levels and low household income groups). In the sensitivity analysis of including participants who answered “about the same”, the results did not change materially either using better as the reference group (Appendix Tables 11) or using “about the same” as the reference group (Appendix Tables 12): people with multiple comorbidities were less likely to report better age-comparative SRH while more likely to report worse age-comparative SRH.

**Table 3:** Multiple comorbidities associated with age-comparative self-rated health status^a^

	Model 1^b^		Model 2^c^		Model 3^d^	
	OR	95% CI	OR	95% CI	OR	95% CI
Cardiometabolic diseases						
Coronary heart disease	5.21	4.91, 5.53	5.23	4.93, 5.56	4.48	4.21, 4.78
Rheumatic heart disease	6.84	5.29, 8.83	6.35	4.91, 8.22	6.17	4.72, 8.06
Stroke	8.06	7.43, 8.75	7.40	6.81, 8.04	7.56	6.93, 8.25
Hypertension	1.46	1.43, 1.49	1.57	1.54, 1.61	1.47	1.44, 1.51
Diabetes	3.12	2.99, 3.25	3.12	2.99, 3.25	3.03	2.89, 3.16
Respiratory diseases						
Tuberculosis	2.06	1.91, 2.21	1.93	1.79, 2.08	1.87	1.73, 2.03
Emphysema/bronchitis	4.50	4.22, 4.79	4.21	3.95, 4.49	4.27	3.99, 4.57
Asthma	4.79	4.18, 5.48	4.53	3.95, 5.19	3.60	3.11, 4.17
Musculoskeletal diseases						
Fracture	1.17	1.13, 1.22	1.21	1.17, 1.26	1.18	1.13, 1.23
Rheumatoid arthritis	2.90	2.72, 3.10	2.95	2.76, 3.16	2.67	2.49, 2.87
Mental diseases						
Depressive Symptoms	4.65	4.11, 5.27	4.52	3.98, 5.12	3.85	3.37, 4.39
Psychiatric disorders	5.73	4.78, 6.86	5.45	4.55, 6.54	4.83	4.00, 5.83
Neurasthenia	4.08	3.73, 4.46	3.99	3.65, 4.37	3.22	2.92, 3.54
Digestive diseases						
Cirrhosis/chronic hepatitis	3.02	2.75, 3.31	2.77	2.52, 3.04	2.73	2.47, 3.01
Peptic ulcer	2.57	2.45, 2.70	2.44	2.33, 2.57	2.47	2.35, 2.60
Gallstone/gallbladder disease	2.36	2.26, 2.45	2.34	2.25, 2.44	2.18	2.09, 2.27
Cancer	6.09	5.25, 7.07	5.17	4.45, 6.01	6.46	5.54, 7.54
Other diseases						
Kidney disease	2.65	2.46, 2.86	2.60	2.41, 2.80	2.29	2.12, 2.48
Head injury	1.56	1.43, 1.70	1.60	1.47, 1.75	1.52	1.38, 1.67

*Abbreviations*: *CI* confidence intervals, *OR* odds ratio

^a^Participants answering “don’t know” (n = 15,691) and “about the same” (n = 321,218) for the age-comparative self-rated health status question were excluded from the analysis, leaving 175,980 participants in this analysis

^b^Model 1: adjusted for age, sex, study location, marital status, education level, income and occupation

^c^Model 2: model 1 plus cigarette smoking, alcohol drinking, physical activity, BMI and family history of five disease (stroke, heart attack, diabetes, mental disorders and cancer)

^d^Model 3: model 2 plus all comorbidities in the same model

From the population perspective, cardiometabolic diseases (hypertension, diabetes, CHD, and stroke), explained the largest part of poor global SRH and worse age-comparative SRH in our study population, followed by emphysema/bronchitis and gallstone/gallbladder disease. Whereas the contribution of rheumatic heart diseases, psychiatric disorders and cancer was small because of the low prevalence (Table 4).

**Table 4:** Population attributable risks for poor self-rated health by multiple comorbidities^a^

	Global SRH (Total *n* = 512,889)		Age-comparative SRH (Total *n* = 175,980)	
	PAR	95% CI	PAR	95% CI
Cardiometabolic diseases				
Coronary heart disease	4.44	4.14, 4.75	9.51	8.84, 10.25
Rheumatic heart disease	0.40	0.30, 0.51	0.92	0.67, 1.25
Stroke	3.20	2.92, 3.46	10.19	9.30, 11.14
Hypertension	7.84	7.26, 8.13	13.79	13.02, 14.78
Diabetes	4.35	4.08, 4.62	10.71	10.05, 11.32
Respiratory diseases				
Tuberculosis	0.71	0.61, 0.83	1.28	1.08, 1.51
Emphysema/bronchitis	3.67	3.43, 3.93	7.81	7.19, 8.46
Asthma	0.57	0.48, 0.68	1.41	1.15, 1.71
Musculoskeletal diseases				
Fracture	0.69	0.48, 0.89	1.23	0.89, 1.56
Rheumatoid arthritis	1.69	1.53, 1.87	3.34	2.99, 3.73
Mental diseases				
Depressive Symptoms	1.05	0.91, 1.20	1.79	1.49, 2.12
Psychiatric disorders	0.43	0.35, 0.52	1.40	1.10, 1.76
Neurasthenia	1.25	1.11, 1.39	2.40	2.09, 2.74
Digestive diseases				
Cirrhosis/chronic hepatitis	0.80	0.70, 0.92	2.05	1.75, 2.37
Peptic ulcer	2.62	2.44, 2.84	5.42	5.00, 5.87
Gallstone/gallbladder disease	3.44	3.22, 3.72	6.65	6.18, 7.12
Cancer	0.77	0.66, 0.90	2.66	2.22, 3.17
Other diseases				
Kidney disease	0.92	0.81, 1.05	1.87	1.63, 2.14
Head injury	0.21	0.13, 0.29	0.57	0.42, 0.73

*Abbreviations*: *CI* confidence intervals, *PAR* population attributable risks

^a^Note that the prevalences of these diseases and symptoms overlap and therefore the population attributable risks cannot be summed up but should be considered as relative

We further examined whether treatment would affect SRH, the results suggested that people with diseases and still on treatment tended to report a poor global SRH and worse age-comparative SRH compared with those having a disease but not receiving treatment (Appendix Table 13). For diabetes and hypertension, shorter duration was associated with less worse SRH, while for other diseases, longer duration was associated with a much worse global and age-comparative SRH (Appendix Table 14). Compared with people without any diseases, the OR for poor global SRH was 1.44 for having one disease, 2.37 and 3.91 for two and three diseases, respectively, and 6.45 when four or more diseases were present (*P* for trend < 0.001; Appendix Table 15). For worse age-comparative SRH, the corresponding OR was 2.13, 5.15, 10.50, and 20.39 for one, two, three and four or more diseases (*P* for trend < 0.001; Appendix Table 15).

When looking at different types of cancer, liver cancer had the greatest impact on global SRH (OR: 3.85; 95% CI: 1.48, 10.01), followed by lung cancer, stomach cancer, other cancer types, intestine cancer, breast cancer, cervix cancer and esophagus cancer. For age-comparative SRH, esophagus cancer had the greatest impact (OR: 8.94; 95% CI: 5.43, 14.74), followed by lung cancer, stomach cancer, other cancer types, liver cancer, breast cancer, intestine cancer and cervix cancer (Appendix Table 16).

When examining the influence of undiagnosed diseases on the association, we found that the association become stronger in the sensitivity analysis of using self-reported hypertension and diabetes compared to the main analysis of using both self-reported and screening-detected (i.e., undiagnosed) hypertension and diabetes (Appendix Table 17).

## Discussion

In this large population-based prospective study, we found that having multiple diseases significantly increased the chance of reporting a poor global SRH and a worse age-comparative SRH, although the magnitude of different diseases varied substantially. We also found cardiometabolic diseases (hypertension, diabetes, CHD and stroke) had the strongest impact on both global SRH and age-comparative SRH at the population level.

Overall, presence of cardiovascular disease (CHD, stroke and rheumatic heart disease) had the biggest impact on global SRH. Other diseases that have a great impact on global SRH including respiratory disease (emphysema/bronchitis and asthma), mental disorders and cancer (adjusted ORs were all above 2.00). Consistent with our findings, Haseli-Mashhadi et al. [20] found that cardiovascular disease had the strongest impact on global SRH in a study of 3289 middle-aged and elderly Chinese adults. Some other studies in different populations also found that cardiovascular disease, cancer and mental diseases were the major determinants of global SRH [19, 21, 28, 29].

In general, the associations between diseases and age-comparative SRH were stronger than the associations with global SRH. Consistently, cardiovascular disease, cancer and mental diseases had the major impact on reporting worse age-comparative SRH. Our findings are consistent with some other studies which found that chronic diseases substantially affected people’s perception of their health status compared to their peers of similar age. In our study, stroke was the most important factor affecting age-comparative SRH among total population, rural residence and male participants. A study in Sweden also found that stroke had the biggest impact on reporting worse age-comparative SRH [18], while a study in Hong Kong found that depressive symptoms was the most significant factor associated with worse age-comparative SRH [17].

Chronic disease like CHD, stroke, rheumatic heart disease, cancer, and emphysema or bronchitis have a great influence on people’s functional ability, which in turn is significantly associated with both global and age-comparative SRH [3, 15, 17]. Meanwhile, people with chronic diseases can experience pain and disability that results in poor SRH. Depression was another important factor affecting both global and age-comparative SRH in our study: people who are more depressed are more inclined to rate their health more negatively, which is consistent with previous studies [30–32].

In our population of middle-aged and elderly men and women, hypertension had the strongest impact on both global SRH and age-comparative SRH at the population level. The contributions of diabetes, CHD and stroke to the burden of poor global SRH and worse age- comparative SRH were also large due to either high prevalence rates or large effect size. However, it should be noted that the population level contribution of different diseases may vary in populations with different prevalence rates.

We found that people with diseases and still on treatment generally reported worse SRH compared to those with a disease but not receiving treatment, which might be because people still on treatment had worse disease status or had more serious conditions. In our study, longer duration was associated with much worse SRH for diabetes and hypertension, and this may be because longer duration of diabetes and hypertension was associated with higher risks of complications, cardiovascular and cerebrovascular diseases [33–35]. In addition, the number of comorbidities was significantly associated with worse SRH, which is in line with the literature [14, 15, 36]. People with more diseases have increased possibility of dysfunction and suffer more pain and inconvenience both physically and mentally, which can lead them to rate their health more negatively.

The major strength of our study is that we have covered a wide range of diseases and the analysis was based on a large population sample of both men and women in ten regions of China. We also included both global and age-comparative SRH measures in our study, and the large sample size allowed us to conduct stratified analyses with sufficient power. Many of the previous studies have only studied the contribution of diseases on global SRH at individual level, whereas we studied the contribution of diseases on both global and age-comparative SRH at both the individual and population level. However, the study has several limitations that should be noted. The main limitation is the cross-sectional design of the study, which does not allow for investigating the temporal associations between various diseases and SRH. Another limitation is that most of the studied diseases were self-reported which may be subject to misclassifications. The sensitivity analysis of using self-reported hypertension and diabetes vs. the main analysis of using both self-reported and screening-detected cases indicated that this type of misclassification might overestimate the association. This is probably because people with self-reported physician-diagnosed diseases were more likely to be severe cases, and thus the association would be attenuated if we included both self-reported and undiagnosed diseases in the analysis. However, the magnitude of attenuation may depend on the proportion of undiagnosed cases. For hypertension and diabetes, the proportion of undiagnosed cases was not negligible, in other words, the awareness of the disease was not very high in the Chinese populations [37, 38]. While for other diseases (such as heart diseases, stroke, cancer etc.), the proportion might be very high and the influence of the undiagnosed diseases would not substantially change our conclusions. In addition, although we have adjusted for a wide range of confounding factors such as socioeconomic, demographic and behavioral variables, residual and unmeasured confounding is still possible. For example, functional ability and/or cognitive levels, which have been previously found to be associated with SRH [3, 15, 39], were not available in our study. Finally, detailed information on treatment and severity status of each disease was not available and more studies are still needed to examine strategies to promote better SRH among people with certain diseases.

## Conclusions

We found chronic physical diseases (such as cardiovascular disease, diabetes, cancer) and mental illnesses are major determinants of poor global SRH and worse age-comparative SRH. Cardiometabolic diseases (hypertension, diabetes, CHD and stroke) had the strongest impact on both global SRH and age-comparative SRH at the population level. Our study suggests that prevention measures concentrated on these conditions would have the largest effect on the total burden of poor SRH and its consequences such as poor quality of life and use of health care services. Knowledge of diseases that have a great impact on both global SRH and age-comparative SRH might help in exploring the factors behind low SRH status. Knowing the patient’s SRH status and the common determinants of SRH could help health professionals in the health care sectors to focus on the patient’s own goals for health and function.

## Appendix

**Table 5:** Multiple comorbidities associated with global self-rated health stratified by residential location and sex

	Administrative region				*P* for interaction	Sex				*P* for interaction
	Rural		Urban			Male		Female		
	OR	95% CI	OR	95% CI		OR	95% CI	OR	95% CI	
Cardiometabolic diseases										
Coronary heart disease	2.75	2.55, 2.96	2.34	2.22, 2.46	< 0.001	2.57	2.41, 2.75	2.50	2.37, 2.64	0.12
Rheumatic heart disease	3.63	2.82, 4.67	2.62	2.03, 3.37	0.07	4.41	2.99, 6.49	2.86	2.33, 3.50	0.07
Stroke	2.92	2.67, 3.20	2.69	2.50, 2.89	0.02	3.16	2.93, 3.40	2.69	2.46, 2.93	0.23
Hypertension	1.15	1.13, 1.17	1.22	1.20, 1.25	0.006	1.24	1.22, 1.27	1.24	1.22, 1.27	0.01
Diabetes	1.64	1.57, 1.71	1.83	1.77, 1.90	0.007	1.90	1.82, 1.98	1.69	1.63, 1.75	0.006
Respiratory diseases										
Tuberculosis	1.60	1.48, 1.73	1.37	1.28, 1.47	< 0.001	1.55	1.45, 1.66	1.40	1.30, 1.51	0.31
Emphysema/bronchitis	2.87	2.71, 3.05	1.81	1.70, 1.92	< 0.001	2.49	2.34, 2.64	2.45	2.31, 2.60	0.99
Asthma	1.89	1.63, 2.20	2.16	1.93, 2.41	0.69	2.05	1.78, 2.35	2.05	1.81, 2.31	0.81
Musculoskeletal diseases										
Fracture	1.09	1.06, 1.13	1.01	0.98, 1.04	< 0.001	1.09	1.05, 1.12	1.10	1.07, 1.14	0.88
Rheumatoid arthritis	1.82	1.70, 1.94	1.91	1.80, 2.03	0.80	1.71	1.58, 1.85	1.87	1.77, 1.97	0.008
Mental diseases										
Depressive Symptoms	2.50	2.24, 2.78	2.49	2.16, 2.89	0.53	2.60	2.22, 3.05	2.70	2.44, 3.00	0.98
Psychiatric disorders	1.94	1.68, 2.24	1.88	1.62, 2.19	0.68	2.40	1.97, 2.93	2.04	1.79, 2.31	0.10
Neurasthenia	2.00	1.82, 2.19	1.91	1.75, 2.07	0.22	1.90	1.70, 2.13	2.23	2.07, 2.41	0.005
Digestive diseases										
Cirrhosis/chronic hepatitis	1.56	1.45, 1.68	1.89	1.74, 2.06	< 0.001	1.70	1.59, 1.83	1.58	1.45, 1.73	0.07
Peptic ulcer	1.80	1.72, 1.89	1.61	1.55, 1.68	< 0.001	1.57	1.50, 1.63	1.89	1.80, 1.99	< 0.001
Gallstone/gallbladder disease	1.57	1.51, 1.62	1.37	1.32, 1.42	< 0.001	1.52	1.45, 1.60	1.61	1.56, 1.66	0.004
Cancer	2.09	1.81, 2.40	2.71	2.37, 3.09	0.01	2.99	2.54, 3.51	2.31	2.05, 2.61	0.02
Other diseases										
Kidney disease	1.75	1.63, 1.88	1.40	1.30, 1.51	< 0.001	1.50	1.38, 1.63	1.72	1.61, 1.84	< 0.001
Head injury	1.26	1.17, 1.37	1.20	1.11, 1.30	0.10	1.19	1.11, 1.28	1.20	1.09, 1.31	0.81

*Abbreviations*: *CI* confidence intervals, *OR* odds ratio

Odds ratios (95% CI) were calculated after adjustment of age, sex, study location, marital status, household income, education level, occupation, alcohol drinking, cigarette smoking, physical activity, BMI, family history of five diseases (stroke, heart attack, diabetes, mental disorders and cancer) and all comorbidities, except for the stratified variable in the corresponding stratified analysis

**Table 6:** Multiple comorbidities associated with global self-rated health stratified by age and educational level

	Age				*P* for interaction	Education level						*P* for interaction
	30-59 yr		≥ 60 yr			No formal education		Primary school		Middle school or higher		
	OR	95% CI	OR	95% CI		OR	95% CI	OR	95% CI	OR	95% CI	
Cardiometabolic diseases												
Coronary heart disease	3.03	2.84,3 .24	2.50	2.37, 2.64	< 0.001	2.42	2.18, 2.70	2.59	2.40, 2.80	2.56	2.42, 2.71	0.79
Rheumatic heart disease	3.06	2.47, 3.78	3.68	2.62, 5.16	0.55	3.12	2.12, 4.58	3.57	2.52, 5.03	3.01	2.34, 3.87	0.71
Stroke	3.33	3.04, 3.65	2.98	2.77, 3.21	< 0.001	3.08	2.68, 3.54	2.92	2.64, 3.23	2.94	2.72, 3.19	0.40
Hypertension	1.27	1.25, 1.29	1.19	1.16, 1.22	< 0.001	1.14	1.11, 1.18	1.21	1.18, 1.23	1.32	1.29, 1.34	< 0.001
Diabetes	1.83	1.76, 1.89	1.71	1.64, 1.79	< 0.001	1.70	1.61, 1.81	1.67	1.59, 1.75	1.88	1.81, 1.96	< 0.001
Respiratory diseases												
Tuberculosis	1.67	1.56, 1.79	1.35	1.25, 1.46	< 0.001	1.70	1.46, 1.98	1.45	1.32, 1.60	1.47	1.37, 1.57	0.10
Emphysema/bronchitis	2.66	2.51, 2.81	2.27	2.13, 2.42	< 0.001	2.79	2.55, 3.04	2.77	2.57, 2.97	2.04	1.91, 2.18	< 0.001
Asthma	2.16	1.93, 2.41	1.92	1.64, 2.25	0.04	1.86	1.51, 2.30	2.02	1.70, 2.42	2.14	1.89, 2.42	0.66
Musculoskeletal diseases												
Fracture	1.10	1.07, 1.13	1.09	1.05, 1.15	0.03	1.25	1.18, 1.31	1.10	1.05, 1.15	1.05	1.02, 1.09	0.06
Rheumatoid arthritis	1.97	1.87, 2.09	1.58	1.47, 1.71	< 0.001	1.83	1.66, 2.02	1.77	1.63, 1.92	1.87	1.75, 2.00	0.58
Mental diseases												
Depressive Symptoms	2.58	2.34, 2.84	2.71	2.20, 3.34	0.93	2.83	2.34, 3.42	2.52	2.17, 2.92	2.67	2.35, 3.05	0.55
Psychiatric disorders	2.28	2.02, 2.58	1.75	1.40, 2.19	0.002	2.34	1.90, 2.88	2.02	1.68, 2.44	2.24	1.90, 2.65	0.39
Neurasthenia	2.42	2.24, 2.61	1.64	1.47, 1.83	< 0.001	2.39	2.07, 2.76	2.13	1.89, 2.41	2.06	1.90, 2.25	0.72
Digestive diseases												
Cirrhosis/chronic hepatitis	1.81	1.70, 1.93	1.40	1.24, 1.57	< 0.001	1.55	1.33, 1.80	1.67	1.51, 1.85	1.72	1.60, 1.86	0.69
Peptic ulcer	1.82	1.76, 1.89	1.40	1.32, 1.49	< 0.001	1.81	1.67, 1.96	1.74	1.64, 1.85	1.63	1.56, 1.70	0.04
Gallstone/gallbladder disease	1.69	1.64, 1.74	1.40	1.33, 1.47	< 0.001	1.64	1.56, 1.73	1.63	1.56, 1.71	1.54	1.48, 1.59	0.12
Cancer	2.79	2.46, 3.17	2.41	2.08, 2.81	0.004	2.70	2.15, 3.40	2.45	2.07, 2.89	2.62	2.28, 3.01	0.73
Other diseases												
Kidney disease	1.70	1.61, 1.81	1.48	1.33, 1.64	< 0.001	1.83	1.58, 2.13	1.68	1.52, 1.85	1.57	1.47, 1.68	0.16
Head injury	1.21	1.13, 1.29	1.04	0.92, 1.18	0.001	1.26	1.07, 1.48	1.24	1.12, 1.37	1.15	1.07, 1.24	0.49

*Abbreviations*: *CI* confidence intervals, *OR* odds ratio

Odds ratios (95% CI) were calculated after adjustment of age, sex, study location, marital status, household income, education level, occupation, alcohol drinking, cigarette smoking, physical activity, BMI, family history of five diseases (stroke, heart attack, diabetes, mental disorders and cancer) and all comorbidities, except for the stratified variable in the corresponding stratified analysis

**Table 7:** Multiple comorbidities associated with global self-rated health stratified by household income

	Household income, RMB						*P* for interaction
	< 10,000		10,000–34,999		≥ 35,000		
	OR	95% CI	OR	95% CI	OR	95% CI	
Cardiometabolic diseases							
Coronary heart disease	2.51	2.29, 2.76	2.53	2.40, 2.68	2.52	2.30, 2.77	0.86
Rheumatic heart disease	2.41	1.81, 3.19	3.80	2.91, 4.97	3.64	2.30, 5.75	0.09
Stroke	3.24	2.89, 3.62	2.79	2.59, 3.01	2.77	2.41, 3.18	0.60
Hypertension	1.19	1.16, 1.22	1.25	1.23, 1.28	1.32	1.28, 1.37	< 0.001
Diabetes	1.71	1.61, 1.81	1.74	1.68, 1.80	1.87	1.77, 1.99	0.001
Respiratory diseases							
Tuberculosis	1.71	1.54, 1.89	1.41	1.32, 1.51	1.41	1.26, 1.58	0.01
Emphysema/bronchitis	2.75	2.53, 2.99	2.39	2.25, 2.54	2.35	2.15, 2.56	0.08
Asthma	1.98	1.62, 2.43	2.05	1.81, 2.31	2.11	1.75, 2.54	0.78
Musculoskeletal diseases							
Fracture	1.14	1.08, 1.20	1.08	1.04, 1.11	1.10	1.05, 1.15	0.04
Rheumatoid arthritis	1.81	1.66, 1.97	1.83	1.72, 1.95	1.85	1.67, 2.06	0.65
Mental diseases							
Depressive Symptoms	2.98	2.56, 3.47	2.55	2.26, 2.87	2.27	1.78, 2.89	0.10
Psychiatric disorders	2.31	1.91, 2.78	2.14	1.84, 2.48	2.08	1.60, 2.70	0.86
Neurasthenia	2.00	1.75, 2.28	2.18	2.00, 2.37	2.25	1.97, 2.56	0.07
Digestive diseases							
Cirrhosis/chronic hepatitis	1.83	1.62, 2.06	1.72	1.59, 1.86	1.51	1.35, 1.68	0.008
Peptic ulcer	1.92	1.80, 2.04	1.66	1.59, 1.74	1.50	1.40, 1.61	< 0.001
Gallstone/gallbladder disease	1.71	1.63, 1.81	1.57	1.51, 1.62	1.52	1.45, 1.60	0.003
Cancer	1.93	1.62, 2.30	2.63	2.31, 3.00	3.92	3.07, 5.01	< 0.001
Other diseases							
Kidney disease	1.71	1.55, 1.88	1.62	1.51, 1.74	1.54	1.36, 1.73	0.16
Head injury	1.35	1.21, 1.50	1.11	1.03, 1.20	1.17	1.03, 1.34	0.02

*Abbreviations*: *CI* confidence intervals, *OR* odds ratio

Odds ratios (95% CI) were calculated after adjustment of age, sex, study location, marital status, household income, education level, occupation, alcohol drinking, cigarette smoking, physical activity, BMI, family history of five diseases (stroke, heart attack, diabetes, mental disorders and cancer) and all comorbidities, except for the stratified variable in the corresponding stratified analysis

**Table 8:** Multiple comorbidities associated with age-comparative self-rated health stratified by residential location and sex^a^

	Administrative region				*P* for interaction	Sex				*P* for interaction
	Rural		Urban			Male		Female		
	OR	95% CI	OR	95% CI		OR	95% CI	OR	95% CI	
Cardiometabolic diseases										
Coronary heart disease	5.62	4.92, 6.43	4.20	3.90, 4.53	< 0.001	4.96	4.47, 5.49	4.24	3.91, 4.60	0.37
Rheumatic heart disease	6.96	4.66, 10.38	5.37	3.74, 7.72	0.29	7.06	4.03, 12.36	5.81	4.29, 7.86	0.66
Stroke	9.40	7.98, 11.06	6.29	5.66, 6.97	< 0.001	10.21	9.08, 11.48	5.50	4.82, 6.26	< 0.001
Hypertension	1.40	1.35, 1.45	1.53	1.48, 1.58	0.001	1.44	1.39, 1.50	1.49	1.45, 1.54	< 0.001
Diabetes	2.82	2.61, 3.05	3.18	3.01, 3.36	0.30	3.41	3.17, 3.66	2.80	2.64, 2.96	0.07
Respiratory diseases										
Tuberculosis	2.23	1.95, 2.56	1.65	1.49, 1.83	< 0.001	1.88	1.68, 2.11	1.82	1.62, 2.06	0.94
Emphysema/bronchitis	6.35	5.71, 7.06	3.16	2.88, 3.46	< 0.001	4.38	3.97, 4.82	4.19	3.81, 4.61	0.99
Asthma	3.24	2.49, 4.21	4.30	3.62, 5.11	0.40	3.57	2.87, 4.44	3.59	2.95, 4.37	0.92
Musculoskeletal diseases										
Fracture	1.23	1.15, 1.31	1.23	1.17, 1.29	0.11	1.18	1.12, 1.26	1.16	1.09, 1.23	0.52
Rheumatoid arthritis	2.76	2.48, 3.08	2.69	2.45, 2.96	0.14	2.37	2.08, 2.69	2.79	2.56, 3.04	0.002
Mental diseases										
Depressive Symptoms	3.45	2.91, 4.09	4.48	3.66, 5.48	0.04	3.77	3.00, 4.75	3.91	3.33, 4.59	0.88
Psychiatric disorders	4.51	3.44,5.90	5.17	3.98, 6.72	0.32	5.65	3.93, 8.13	4.48	3.59, 5.58	0.29
Neurasthenia	3.35	2.88,3.90	3.16	2.79, 3.57	0.25	2.61	2.19, 3.11	3.50	3.12, 3.93	0.001
Digestive diseases										
Cirrhosis/chronic hepatitis	3.00	2.61, 3.44	2.85	2.48, 3.28	0.96	3.04	2.68, 3.46	2.28	1.95, 2.66	0.004
Peptic ulcer	3.15	2.91, 3.40	2.09	1.95, 2.23	< 0.001	2.21	2.06, 2.37	2.82	2.61, 3.05	< 0.001
Gallstone/gallbladder disease	2.37	2.22, 2.53	2.10	1.99, 2.22	< 0.001	2.02	1.86, 2.19	2.20	2.09, 2.31	0.001
Cancer	5.10	3.98, 6.53	7.20	5.92, 8.76	0.07	8.67	6.62, 11.35	5.33	4.41, 6.44	0.007
Other diseases										
Kidney disease	2.36	2.11, 2.63	2.32	2.07, 2.60	0.98	2.18	1.91, 2.50	2.38	2.15, 2.63	0.09
Head injury	1.59	1.39, 1.83	1.47	1.30, 1.66	0.83	1.45	1.29, 1.64	1.62	1.39, 1.88	0.28

*Abbreviations*: *CI* confidence intervals, *OR* odds ratio

^a^Participants answering “don’t know” (n = 15,691) and “about the same” (n = 321,218) for the age-comparative self-rated health status question were excluded from the analysis, leaving 175,980 participants in this analysis.

Odds ratios (95% CI) were calculated after adjustment of age, sex, study location, marital status, household income, education level, occupation, alcohol drinking, cigarette smoking, physical activity, BMI, family history of five diseases (stroke, heart attack, diabetes, mental disorders and cancer) and all comorbidities, except for the stratified variable in the corresponding stratified analysis

**Table 9:** Multiple comorbidities associated with age-comparative self-rated health stratified by age and educational level^a^

	Age				*P* for interaction	Education level						*P* for interaction
	30-59 yr		≥ 60 yr			No formal education		Primary school		Middle school or higher		
	OR	95%CI	OR	95% CI		OR	95% CI	OR	95% CI			
Cardiometabolic diseases												
Coronary heart disease	6.55	5.89, 7.29	4.01	3.68, 4.38	< 0.001	3.48	2.92, 4.14	5.32	4.68, 6.06	4.68	4.30, 5.08	0.03
Rheumatic heart disease	6.31	4.55, 8.74	5.21	3.24, 8.39	0.56	5.78	3.05, 10.97	4.69	2.96, 7.43	7.71	5.27, 11.28	0.39
Stroke	9.54	8.22, 11.07	7.92	7.07, 8.88	< 0.001	8.04	6.33, 10.22	8.02	6.81, 9.43	7.96	7.09, 8.94	0.78
Hypertension	1.42	1.38, 1.46	1.29	1.23, 1.35	0.07	1.30	1.24, 1.37	1.40	1.34, 1.47	1.57	1.52, 1.63	0.007
Diabetes	3.19	3.01, 3.39	2.68	2.49, 2.88	< 0.001	2.60	2.34, 2.88	2.79	2.56, 3.03	3.42	3.21, 3.63	< 0.001
Respiratory diseases												
Tuberculosis	2.34	2.10, 2.61	1.46	1.28, 1.66	< 0.001	2.52	1.96, 3.25	1.83	1.56, 2.15	1.85	1.66, 2.05	0.03
Emphysema/bronchitis	4.63	4.22, 5.08	3.67	3.31, 4.07	0.001	4.37	3.76, 5.07	5.16	4.59, 5.81	3.48	3.14, 3.85	< 0.001
Asthma	3.96	3.30, 4.74	3.18	2.47, 4.09	0.06	3.67	2.39, 5.64	2.63	1.98, 3.50	4.26	3.55, 5.13	0.15
Musculoskeletal diseases												
Fracture	1.18	1.13, 1.24	1.12	1.03, 1.22	0.003	1.33	1.21, 1.46	1.11	1.03, 1.20	1.18	1.11, 1.25	0.38
Rheumatoid arthritis	3.04	2.78, 3.32	1.98	1.75, 2.24	< 0.001	2.42	2.06, 2.85	2.67	2.35, 3.04	2.81	2.54, 3.10	0.67
Mental diseases												
Depressive Symptoms	4.01	3.46, 4.65	3.13	2.33, 4.22	0.02	3.66	2.70, 4.95	3.62	2.83, 4.61	4.17	3.48, 5.01	0.25
Psychiatric disorders	5.68	4.53, 7.11	3.46	2.39, 5.01	0.002	4.34	2.97, 6.35	3.88	2.80, 5.38	5.82	4.37, 7.77	0.07
Neurasthenia	4.07	3.61, 4.58	1.89	1.59, 2.25	< 0.001	3.71	2.85, 4.85	2.94	2.43, 3.57	3.25	2.88, 3.67	0.45
Digestive diseases												
Cirrhosis/chronic hepatitis	3.16	2.82, 3.55	2.13	1.75, 2.60	< 0.001	2.68	2.00, 3.59	2.86	2.36, 3.48	2.71	2.39, 3.07	0.80
Peptic ulcer	2.74	2.57, 2.91	1.85	1.67, 2.04	< 0.001	2.70	2.34, 3.10	2.57	2.34, 2.83	2.40	2.24, 2.56	0.07
Gallstone/gallbladder disease	2.37	2.25, 2.50	1.68	1.55, 1.82	< 0.001	2.26	2.05, 2.48	2.17	2.00, 2.36	2.14	2.01, 2.27	0.04
Cancer	7.69	6.19, 9.56	5.38	4.28, 6.76	0.002	4.48	3.13, 6.41	6.96	5.19, 9.34	7.16	5.80, 8.85	0.04
Other diseases												
Kidney disease	2.49	2.27, 2.73	1.92	1.63, 2.26	< 0.001	2.07	1.64, 2.61	2.19	1.89, 2.55	2.40	2.17, 2.66	0.35
Head injury	1.54	1.39, 1.71	1.44	1.16, 1.77	0.06	1.70	1.29, 2.22	1.65	1.39, 1.96	1.42	1.26, 1.60	0.42

*Abbreviations*: *CI* confidence intervals, *OR* odds ratio

^a^Participants answering “don’t know” (*n* = 15,691) and “about the same” (*n* = 321,218) for the age-comparative self-rated health status question were excluded from the analysis, leaving 175,980 participants in this analysis

Odds ratios (95% CI) were calculated after adjustment of age, sex, study location, marital status, household income, education level, occupation, alcohol drinking, cigarette smoking, physical activity, BMI, family history of five diseases (stroke, heart attack, diabetes, mental disorders and cancer) and all comorbidities, except for the stratified variable in the corresponding stratified analysis

**Table 10:** Multiple comorbidities associated with age-comparative self-rated health stratified by household income^a^

	Household income, RMB						*P* for interaction
	< 10,000		10,000–34,999		≥ 35,000		
	OR	95% CI	OR	95% CI	OR	95% CI	
Cardiometabolic diseases							
Coronary heart disease	4.47	3.88, 5.16	4.56	4.20, 4.96	4.36	3.77, 5.04	0.37
Rheumatic heart disease	4.61	3.07, 6.92	6.33	4.30, 9.30	14.13	6.12, 32.62	0.08
Stroke	8.32	7.00, 9.88	7.32	6.53, 8.21	7.29	5.87, 9.05	0.97
Hypertension	1.35	1.29, 1.41	1.50	1.45, 1.55	1.69	1.58, 1.79	< 0.001
Diabetes	2.67	2.44, 2.91	3.04	2.87, 3.23	3.41	3.07, 3.78	< 0.001
Respiratory diseases							
Tuberculosis	2.40	2.05, 2.81	1.71	1.52, 1.91	1.68	1.38, 2.04	< 0.001
Emphysema/bronchitis	4.60	4.07, 5.20	4.22	3.83, 4.65	3.89	3.35, 4.51	0.04
Asthma	2.78	2.05, 3.77	3.82	3.14, 4.65	4.19	3.08, 5.71	0.19
Musculoskeletal diseases							
Fracture	1.19	1.09, 1.30	1.17	1.11, 1.24	1.18	1.08, 1.29	0.69
Rheumatoid arthritis	2.36	2.08, 2.68	2.90	2.62, 3.20	2.58	2.17, 3.07	0.06
Mental diseases							
Depressive Symptoms	4.12	3.33, 5.10	3.64	3.02, 4.39	4.16	2.84, 6.07	0.72
Psychiatric disorders	4.32	3.17, 5.88	4.86	3.72, 6.36	6.75	4.07, 11.18	0.15
Neurasthenia	2.79	2.28, 3.41	3.32	2.92, 3.77	3.55	2.89, 4.36	0.06
Digestive diseases							
Cirrhosis/chronic hepatitis	2.50	2.08, 2.99	2.88	2.51, 3.32	2.81	2.26, 3.49	0.57
Peptic ulcer	2.72	2.47, 3.00	2.42	2.26, 2.60	2.23	1.98, 2.51	0.04
Gallstone/gallbladder disease	2.26	2.09, 2.45	2.09	1.96, 2.21	2.31	2.10, 2.53	0.03
Cancer	5.30	3.98, 7.08	6.21	5.05, 7.63	10.32	7.03, 15.15	0.02
Other diseases							
Kidney disease	2.07	1.81, 2.37	2.48	2.21, 2.78	2.31	1.89, 2.82	0.31
Head injury	1.56	1.32, 1.84	1.54	1.35, 1.75	1.36	1.08, 1.71	0.71

*Abbreviations*: *CI* confidence intervals, *OR* odds ratio

^a^Participants answering “don’t know” (*n* = 15,691) and “about the same” (n = 321,218) for the age-comparative self-rated health status question were excluded from the analysis, leaving 175,980 participants in this analysis

Odds ratios (95% CI) were calculated after adjustment of age, sex, study location, marital status, household income, education level, occupation, alcohol drinking, cigarette smoking, physical activity, BMI, family history of five diseases (stroke, heart attack, diabetes, mental disorders and cancer) and all comorbidities, except for the stratified variable in the corresponding stratified analysis

**Table 11:** Multiple comorbidities associated with age-comparative self-rated health status: multinomial logistic regression analysis using “better” as the reference group^a^

	Better	About the same		Worse	
	OR	OR	95% CI	OR	95% CI
Cardiometabolic diseases					
Coronary heart disease	1	1.65	1.56, 1.75	4.44	4.17, 4.72
Rheumatic heart disease	1	1.52	1.17, 1.98	5.97	4.59, 7.76
Stroke	1	1.80	1.66, 1.96	7.42	6.82, 8.07
Hypertension	1	1.18	1.16, 1.20	1.48	1.45, 1.51
Diabetes	1	1.44	1.39, 1.50	2.99	2.87, 3.12
Respiratory diseases					
Tuberculosis	1	1.17	1.09, 1.25	1.89	1.75, 2.04
Emphysema/bronchitis	1	1.49	1.40, 1.58	4.35	4.07, 4.64
Asthma	1	1.36	1.19, 1.56	3.43	2.98, 3.94
Musculoskeletal diseases					
Fracture	1	0.99	0.96, 1.02	1.18	1.14, 1.23
Rheumatoid arthritis	1	1.26	1.18, 1.34	2.65	2.48, 2.84
Mental diseases					
Depressive Symptoms	1	1.06	0.94, 1.21	3.71	3.26, 4.21
Psychiatric disorders	1	1.80	1.51, 2.16	4.72	3.93, 5.67
Neurasthenia	1	1.35	1.24, 1.47	3.22	2.94, 3.54
Digestive diseases					
Cirrhosis/chronic hepatitis	1	1.33	1.22, 1.45	2.63	2.39, 2.89
Peptic ulcer	1	1.36	1.30, 1.42	2.48	2.36, 2.61
Gallstone/gallbladder disease	1	1.30	1.26, 1.35	2.16	2.07, 2.25
Cancer	1	1.64	1.41, 1.90	6.44	5.54, 7.49
Other diseases					
Kidney disease	1	1.27	1.18, 1.36	2.36	2.19, 2.55
Head injury	1	1.03	0.96, 1.11	1.53	1.40, 1.67

*Abbreviations*: *CI* confidence intervals, *OR* odds ratio

^a^Participants answering “don’t know” (*n* = 15,691) for the age-comparative self-rated health status question were excluded from the analysis, leaving 497,198 participants in this analysis

Odds ratios (95% CI) were calculated after adjustment of age, sex, study location, marital status, household income, education level, occupation, alcohol drinking, cigarette smoking, physical activity, BMI, family history of five diseases (stroke, heart attack, diabetes, mental disorders and cancer) and all comorbidities

**Table 12:** Multiple comorbidities associated with age-comparative self-rated health status: multinomial logistic regression analysis using “about the same” as the reference group^a^

	About the same	Better		Worse	
	OR	OR	95% CI	OR	95% CI
Cardiometabolic diseases					
Coronary heart disease	1	0.61	0.57, 0.64	2.69	2.59, 2.80
Rheumatic heart disease	1	0.66	0.51, 0.85	3.92	3.38, 4.55
Stroke	1	0.56	0.51, 0.60	4.12	3.92, 4.33
Hypertension	1	0.85	0.83, 0.86	1.25	1.23, 1.28
Diabetes	1	0.70	0.67, 0.72	2.08	2.02, 2.14
Respiratory diseases					
Tuberculosis	1	0.86	0.80, 0.92	1.62	1.53, 1.72
Emphysema/bronchitis	1	0.67	0.63, 0.72	2.93	2.81, 3.05
Asthma	1	0.74	0.64, 0.84	2.52	2.30, 2.75
Musculoskeletal diseases					
Fracture	1	1.01	0.98, 1.04	1.19	1.15, 1.23
Rheumatoid arthritis	1	0.79	0.75, 0.84	2.10	2.00, 2.21
Mental diseases					
Depressive Symptoms	1	0.94	0.83, 1.07	3.49	3.22, 3.78
Psychiatric disorders	1	0.56	0.46, 0.66	2.62	2.36, 2.91
Neurasthenia	1	0.74	0.68, 0.81	2.39	2.25, 2.55
Digestive diseases					
Cirrhosis/chronic hepatitis	1	0.75	0.69, 0.82	1.97	1.85, 2.10
Peptic ulcer	1	0.74	0.71, 0.77	1.83	1.76, 1.90
Gallstone/gallbladder disease	1	0.77	0.74, 0.80	1.61	1.61, 1.71
Cancer	1	0.61	0.53, 0.71	3.93	3.60, 4.29
Other diseases					
Kidney disease	1	0.79	0.74, 0.85	1.86	1.76, 1.97
Head injury	1	0.97	0.90, 1.05	1.49	1.39, 1.60

*Abbreviations*: *CI* confidence intervals, *OR* odds ratio

^a^Participants answering “don’t know” (*n* = 15,691) for the age-comparative self-rated health status question were excluded from the analysis, leaving 497,198 participants in this analysis

Odds ratios (95% CI) were calculated after adjustment of age, sex, study location, marital status, household income, education level, occupation, alcohol drinking, cigarette smoking, physical activity, BMI, family history of five diseases (stroke, heart attack, diabetes, mental disorders and cancer) and all comorbidities

**Table 13:** Treatment associated with self-rated health status

	Global SRH (Total *n* = 512,889)		Age-comparative SRH (Total *n* = 175,980)	
	OR	95% CI	OR	95% CI
Cardiometabolic diseases				
Coronary heart disease				
Yes, but no treatment now	1.85	1.75, 1.96	2.79	2.55, 3.06
Yes, still on treatment	2.91	2.73, 3.09	5.32	4.85, 5.83
Rheumatic heart disease				
Yes, but no treatment now	2.27	1.84, 2.80	3.67	2.68, 5.05
Yes, still on treatment	7.33	5.01, 10.72	19.27	10.65, 34.87
Stroke				
Yes, but no treatment now	2.12	1.96, 2.28	4.25	3.78, 4.77
Yes, still on treatment	3.11	2.85, 3.39	9.53	8.27, 10.98
Hypertension				
Yes, but no treatment now	1.11	1.09, 1.12	1.16	1.13, 1.19
Yes, still on treatment	1.93	1.89, 1.98	3.38	3.24, 3.53
Diabetes				
Yes, but no treatment now	1.23	1.19, 1.27	1.51	1.42, 1.60
Yes, still on treatment (No insulin)	2.88	2.75, 3.03	6.51	6.00, 7.06
Yes, still on treatment (Use insulin)	3.81	3.36, 4.32	10.01	8.24, 12.16
Respiratory diseases				
TB				
Yes, but no treatment now	1.44	1.37, 1.52	1.82	1.67, 1.98
Yes, still on treatment	3.25	2.42, 4.36	7.53	4.49, 12.62
Emphysema/bronchitis				
Yes, but no treatment now	2.08	1.98, 2.18	3.34	3.09, 3.62
Yes, still on treatment	4.02	3.69, 4.38	8.10	7.08, 9.26
Asthma				
Yes, but no treatment now	1.59	1.43, 1.77	2.42	2.02, 2.89
Yes, still on treatment	3.69	3.06, 4.46	8.21	6.14, 10.97
Musculoskeletal diseases				
Fracture				
Yes, but no treatment now	1.10	1.07, 1.12	1.18	1.13, 1.23
Yes, still on treatment	1.27	1.13, 1.43	1.75	1.43, 2.13
Rheumatoid arthritis				
Yes, but no treatment now	1.63	1.54, 1.71	2.19	2.02, 2.38
Yes, still on treatment	2.64	2.40, 2.90	4.90	4.23, 5.67
Mental diseases				
Psychiatric disorders				
Yes, but no treatment now	1.72	1.50, 1.97	3.34	2.64, 4.23
Yes, still on treatment	3.00	2.53, 3.56	8.95	6.40, 12.52
Neurasthenia				
Yes, but no treatment now	1.90	1.77, 2.03	2.69	2.42, 2.99
Yes, still on treatment	3.49	2.99, 4.07	6.93	5.39, 8.90
Digestive diseases				
Cirrhosis/chronic hepatitis				
Yes, but no treatment now	1.53	1.44, 1.62	2.25	2.02, 2.50
Yes, still on treatment	3.92	3.25, 4.73	13.28	9.25, 19.06
Peptic ulcer				
Yes, but no treatment now	1.52	1.47, 1.58	2.08	1.97, 2.20
Yes, still on treatment	2.82	2.61, 3.05	5.57	4.90, 6.32
Gallstone/gallbladder disease				
Yes, but no treatment now	1.51	1.47, 1.56	2.03	1.94, 2.13
Yes, still on treatment	2.09	1.95, 2.24	3.22	2.87, 3.60
Cancer				
Yes, but no treatment now	2.16	1.94, 2.40	5.70	4.80, 6.78
Yes, still on treatment	5.30	4.20, 6.68	13.72	9.48, 19.86
Other diseases				
Kidney disease				
Yes, but no treatment now	1.44	1.36, 1.52	1.93	1.77, 2.11
Yes, still on treatment	3.21	2.76, 3.74	4.80	3.85, 5.98
Head injury				
Yes, but no treatment now	1.17	1.11, 1.24	1.49	1.35, 1.63
Yes, still on treatment	2.27	1.62, 3.17	7.72	4.08, 14.62

*Abbreviations*: *CI* confidence intervals, *OR* odds ratio

The reference group are those who not suffering from the disease

Odds ratios (95% CI) were calculated after adjustment of age, sex, study location, marital status, household income, education level, occupation, alcohol drinking, cigarette smoking, physical activity, BMI, family history of five diseases (stroke, heart attack, diabetes, mental disorders and cancer) and all comorbidities

Depressive Symptoms is not presented because the diseases is diagnosed on this investigation

**Table 14:** Disease diagnosis time associated with self-rated health status

	Global SRH (Total *n* = 512,889)		Age-comparative SRH (Total *n* = 175,980)	
	OR	95% CI	OR	95% CI
Cardiometabolic diseases				
Coronary heart disease				
Yes, diagnosis time < 10 years	2.67	2.53, 2.81	5.07	4.69, 5.49
Yes, diagnosis time ≥ 10 years	2.02	1.88, 2.17	2.83	2.54, 3.15
Rheumatic heart disease				
Yes, diagnosis time < 10 years	4.31	3.03, 6.13	9.71	5.63, 16.75
Yes, diagnosis time ≥ 10 years	2.88	2.34, 3.55	5.28	3.88, 7.19
Stroke				
Yes, diagnosis time < 10 years	3.08	2.89, 3.29	7.99	7.25, 8.82
Yes, diagnosis time ≥ 10 years	1.89	1.67, 2.14	4.51	3.75, 5.43
Hypertension				
Yes, diagnosis time < 10 years	1.21	1.19, 1.22	1.39	1.35, 1.42
Yes, diagnosis time ≥ 10 years	1.89	1.82, 1.97	3.04	2.85, 3.25
Diabetes				
Yes, diagnosis time < 10 years	1.67	1.62, 1.71	2.71	2.59, 2.84
Yes, diagnosis time ≥ 10 years	2.99	2.72, 3.29	6.69	5.77, 7.76
Respiratory diseases				
TB				
Yes, diagnosis time < 10 years	2.22	1.97, 2.50	3.94	3.20, 4.84
Yes, diagnosis time ≥ 10 years	1.35	1.28, 1.43	1.61	1.47, 1.76
Emphysema/bronchitis				
Yes, diagnosis time < 10 years	2.78	2.60, 2.97	5.33	4.79, 5.93
Yes, diagnosis time ≥ 10 years	2.28	2.16, 2.41	3.69	3.38, 4.02
Asthma				
Yes, diagnosis time < 10 years	2.57	2.16, 3.07	4.12	3.18, 5.34
Yes, diagnosis time ≥ 10 years	1.89	1.70, 2.10	3.43	2.87, 4.09
Musculoskeletal diseases				
Fracture				
Yes, diagnosis time < 10 years	1.12	1.09, 1.16	1.25	1.19, 1.33
Yes, diagnosis time ≥ 10 years	1.08	1.05, 1.12	1.13	1.06, 1.19
Rheumatoid arthritis				
Yes, diagnosis time < 10 years	1.84	1.75, 1.92	2.70	2.51, 2.90
Mental diseases				
Psychiatric disorders				
Yes, diagnosis time < 10 years	2.54	2.12, 3.05	6.91	4.86, 9.81
Yes, diagnosis time ≥ 10 years	1.99	1.75, 2.27	4.20	3.35, 5.26
Neurasthenia				
Yes, diagnosis time < 10 years	2.73	2.43, 3.06	4.87	4.08, 5.82
Yes, diagnosis time ≥ 10 years	1.91	1.78, 2.06	2.64	2.36, 2.97
Digestive diseases				
Cirrhosis/chronic hepatitis				
Yes, diagnosis time < 10 years	2.10	1.91, 2.32	3.95	3.32, 4.68
Yes, diagnosis time ≥ 10 years	1.49	1.39, 1.60	2.23	1.97, 2.52
Peptic ulcer				
Yes, diagnosis time < 10 years	1.96	1.87, 2.06	3.29	3.04, 3.57
Yes, diagnosis time ≥ 10 years	1.51	1.45, 1.57	2.03	1.90, 2.17
Gallstone/gallbladder disease				
Yes, diagnosis time < 10 years	1.66	1.61, 1.71	2.36	2.23, 2.48
Yes, diagnosis time ≥ 10 years	1.46	1.40, 1.52	1.88	1.76, 2.01
Cancer				
Yes, diagnosis time < 10 years	3.06	2.72, 3.45	8.60	7.05, 10.48
Yes, diagnosis time ≥ 10 years	1.76	1.49, 2.08	3.57	2.76, 4.61
Other diseases				
Kidney disease				
Yes, diagnosis time < 10 years	1.75	1.64, 1.87	2.36	2.14, 2.61
Yes, diagnosis time ≥ 10 years	1.44	1.33, 1.56	2.13	1.87, 2.43
Head injury				
Yes, diagnosis time < 10 years	1.23	1.13, 1.34	1.56	1.35, 1.80
Yes, diagnosis time ≥ 10 years	1.16	1.08, 1.25	1.52	1.35, 1.72

*Abbreviations*: *CI* confidence intervals, *OR* odds ratio

The reference group are those who not suffering from the disease

Odds ratios (95% CI) were calculated after adjustment of age, sex, study location, marital status, household income, education level, occupation, alcohol drinking, cigarette smoking, physical activity, BMI family history of five diseases (stroke, heart attack, diabetes, mental disorders and cancer) and all comorbidities

Depressive Symptoms is not presented because the disease is diagnosed on this investigation

**Table 15:** The number of diseases associated with self-rated health status

	Global SRH (Total *n* = 512,889)		Age-comparative SRH (Total n = 175,980)	
	OR	95% CI	OR	95% CI
Number of diseases				
One disease	1.44	1.42, 1.46	2.13	2.07, 2.18
Two diseases	2.37	2.32, 2.42	5.15	4.98, 5.33
Three diseases	3.91	3.76, 4.07	10.50	9.88, 11.16
Four or more diseases	6.45	5.96, 6.98	20.39	18.30, 22.71

*Abbreviations*: *CI* confidence intervals, *OR* odds ratio

The reference group are those who not suffering from the disease

Odds ratios (95% CI) were calculated after adjustment of age, sex, study location, marital status, household income, education level, occupation, alcohol drinking, cigarette smoking, physical activity, BMI and family history of five diseases (stroke, heart attack, diabetes, mental disorders and cancer)

**Table 16:** The site of cancer associated with self-rated health status

	Global SRH (Total *n* = 512,889)		Age-comparative SRH (Total *n* = 175,980)	
	OR	95% CI	OR	95% CI
Cancer site				
lung	3.38	2.15, 5.33	8.31	4.08, 16.96
esophagus	2.26	1.71, 3.00	8.94	5.43, 14.74
stomach	2.94	2.17, 4.00	7.79	4.72, 12.86
liver	3.85	1.48, 10.01	7.15	2.28, 22.41
intestine	2.35	1.78, 3.10	5.66	3.59, 8.94
breast	2.31	1.90, 2.81	5.94	4.34, 8.14
cervix	2.30	1.78, 2.99	3.70	2.45, 5.59
other	2.94	2.40, 3.61	7.38	5.41, 10.07

*Abbreviations*: *CI* confidence intervals, *OR* odds ratio

The reference group are those who not suffering from this cancer

Odds ratios (95% CI) were calculated after adjustment of age, sex, study location, marital status, household income, education level, occupation, alcohol drinking, cigarette smoking, physical activity, BMI, family history of five disease (stroke, heart attack, diabetes, mental disorders and cancer) and all comorbidities

**Table 17:** Multiple comorbidities associated with global and age-comparative self-rated health status: sensitivity analysis of using self-reported hypertension and diabetes

	Global SRH (Total *n* = 512,889)		Age-comparative SRH (Total *n* = 175,980)	
	OR	95% CI	OR	95% CI
Main analysis				
Hypertension	1.25	1.23, 1.26	1.47	1.44, 1.51
Diabetes	1.77	1.72, 1.82	3.03	2.89, 3.16
Sensitivity analysis				
Hypertension	1.83	1.79, 1.86	3.08	2.97, 3.20
Diabetes	2.80	2.69, 2.92	6.07	5.67, 6.50

*Abbreviations*: *CI* confidence intervals, *OR* odds ratio

Odds ratios (95% CI) were calculated after adjustment of age, sex, study location, marital status, household income, education level, occupation, alcohol drinking, cigarette smoking, physical activity, BMI, family history of five disease (stroke, heart attack, diabetes, mental disorders and cancer) and all comorbidities

## Abbreviations

BMI: Body mass index
CHD: Coronary heart disease
CI: Confidence intervals
CIDI-SF: Composite International Diagnostic Inventory — short form
CKB: China Kadoorie Biobank
METs: Metabolic equivalent tasks
OR: Odds ratio
PARs: Population attributable risks
SRH: Self-rated health
